# Integrated Analysis of Thyroid Cancer Public Datasets Reveals Role of Post-Transcriptional Regulation on Tumor Progression by Targeting of Immune System Mediators

**DOI:** 10.1371/journal.pone.0141726

**Published:** 2015-11-04

**Authors:** Murilo V. Geraldo, Edna T. Kimura

**Affiliations:** 1 Department of Cell and Developmental Biology, Institute of Biomedical Sciences, University of Sao Paulo, Sao Paulo, Brazil; 2 Department of Structural and Functional Biology, Institute of Biology, State University of Campinas, Campinas, Brazil; University of Connecticut Health Center, UNITED STATES

## Abstract

Papillary thyroid carcinoma (PTC) is a well-differentiated thyroid tumor that accounts for approximately 80% of thyroid cancer cases. On other hand, anaplastic thyroid carcinoma (ATC) is a less frequent, but aggressive subtype, with poor prognosis. MicroRNAs (miRNAs), small non-coding RNAs, have emerged as potent post-transcriptional regulators of gene expression, which modulate the expression of cancer-related genes. Computational analyses estimate that a single miRNA may modulate hundreds of mRNA targets and, at the same time, cooperate with others to regulate one single mRNA transcript. Due to the large number of predicted targets and possible interactions, only a small number of miRNAs have characterized biological roles, and the panorama of miRNA-mediated regulation in thyroid cancer remains to be understood. Taking into consideration the large amount of gene expression data deposited in public databases we aligned miRNA target prediction and gene expression data from public PTC and ATC datasets to construct a network of post-transcriptional regulation in thyroid cancer. After a gene set enrichment analysis we identified signaling pathways and biological processes potentially modulated by the miRNAs deregulated in PTC and ATC. Our results show miRNA-mRNA interaction that could contribute with the de-regulation of key tumor-host mediators, such as extra-cellular matrix molecules, interleukins and interleukin receptors, which could drive a more aggressive behavior and tumor progression. Moreover, our analysis through The Cancer Genome Atlas (TCGA) database revealed that aberrant expression of ECM and cytokines genes is frequent in PTC and is associated with aggressive behavior and decreased overall survival rate. In conclusion, we shed light on the post-transcriptional regulation of gene expression in differentiated and undifferentiated thyroid cancers, revealing a potential role of miRNAs in modulation of tumor-host interaction molecules, particularly ECM molecules and immune system mediators, which could stimulate crosstalk between tumors and the immune system to generate a more aggressive behavior. We propose a novel putative miRNA:mRNA network that could lead to a new path toward functional studies.

## Introduction

In the last decade, increasing numbers of thyroid cancer cases have been observed worldwide. More than 62,000 new cases of thyroid cancer are estimated for 2014, leading to 1,890 deaths [1]. The most prevalent thyroid carcinoma is papillary thyroid carcinoma (PTC), accounting for 80% of the cases, which presents good prognosis. A minority of cases, however, comprise undifferentiated anaplastic thyroid carcinomas (ATCs), with aggressive behavior and poor prognosis.

At present, little is known about the molecular mechanisms orchestrating the loss of differentiation observed in the most aggressive carcinomas. The observation that the mutation T1799A in the *BRAF* coding sequence, the most common genetic alteration in PTC, is also frequently observed in ATC (Nikiforov YE 2004) corroborates the hypothesis that undifferentiated carcinoma arise from previous well-differentiated ones. This genetic alteration has now been explored as a prognostic marker and therapeutic target [2–8].

Several studies have reported aberrant expression of miRNAs, 19–21 nucleotide-long RNA molecules that post-transcriptionally regulate gene expression, in follicular cell-derived thyroid tumors [9–12]. The aberrant expression of these molecules has emerged as a potential diagnostic tool, able to distinguish different subtypes of thyroid cancer from non-tumor tissue and serve as serum markers for monitoring PTC recurrence [13–17]. Although much is known regarding the utility of these molecules as diagnostic and prognostic tools, only a few miRNAs have well characterized functional roles. The identification of miRNA targets is a difficult task, mainly due to the enormous amount of possible interactions between each miRNA and its respective targets, and the possible cooperation among several miRNAs to regulate a single pathway. Thus, the real contribution of multiple deregulated miRNAs to post-transcriptional gene regulation in thyroid oncogenesis and progression, remains unclear. In this context, the combination of bioinformatic tools and online available gene expression data may provide relevant information regarding the biological processes and signaling pathways affected by aberrant post-transcriptional regulation in thyroid cancer. Although the quantity of archived and curated gene expression data in public repositories has increased substantially over the past decade, most of it remains unexplored [18].

In this study, we analyzed a network of predicted targets of the most deregulated miRNAs in PTC and ATC, to improve the identification of novel targets for functional analysis. Combining the literature information with gene expression datasets and gene set enrichment analysis (GSEA) we observed the relationship between microRNA and mRNA expression related with pathways and biological processes affected by the most frequently described deregulated miRNAs in thyroid tumors.

Our analysis shows that miRNA-mediated modulation could affect members of pathways already reported in PTC and ATC, such as MAPK and p53, as well as not yet described miRNA:mRNA interactions, that could implicate miRNAs in post-transcriptional regulation of immune response to promote aggressive behavior and tumor progression.

## Materials and Methods

### miRNA selection

We performed a critical review of the literature using the search terms “miRNA” and “thyroid cancer”. The criteria for selection of studies were: (i) Studies profiling the expression of at least 5 miRNAs; (ii) human thyroid follicular cell-derived tumors; (iii) comparison of tumors with non-tumor thyroid tissue; (iv) fresh, frozen or FFPE samples. Studies comprising non-human thyroid models, thyroid cancer cell lines, and comparison of tumor tissue with benign lesions, as well as fine needle aspiration biopsies (FNABs) as the only source of nucleic acid, were not considered for network construction. Non-English literature was also excluded from the analysis.

To make a more reliable comparison with *Gene Expression Omnibus* (GEO) datasets, miRNA differential expression of PTC variants (follicular variant PTC, tall cell PTC, etc.) were not selected. The nomenclature of miRNAs was corrected according to miRBase (www.miRBase.org, release 19.0). The differentially expressed miRNAs for each thyroid tumor type (PTC and ATC), in each study, were filtered, and those with concordant expression pattern between three or more studies were selected for network construction.

### Computational prediction of miRNA targets

To generate a list of predicted targets of the selected miRNAs, we used the miRWalk webtool (http://www.umm.uni-heidelberg.de/apps/zmf/mirwalk/), which compares eight different computational algorithms for miRNA-target predictions. To increase stringency, we required interactions of a given miRNA with target genes acceptable only if it was reproducibly predicted by at least five algorithms.

### Gene expression datasets

Publicly available microarray data from patients with thyroid cancer was obtained from the *Gene Expression Omnibus* (GEO, http://www.ncbi.nlm.nih.gov/geo/). Differential gene expression between tumor and non-tumor tissue was calculated using the GEO2R program. The GEO database was searched for studies comprising tumor samples from classic PTC and ATC, as well as non-tumor thyroid tissue. For each gene, differential expression was considered valid when concordant expression patterns, with adjusted p-values<0.05, were observed in half or more of the datasets. Due to the lack of information regarding the genetic background of all the tumor samples used for miRNA expression analysis, the status of *BRAF* and *RAS* mutations, as well as RET/PTC rearrangements, could not be incorporated into the dataset analyses.

### Construction of regulatory networks

We aimed to identify anti-correlated miRNA/mRNA expression patterns, i.e., upregulated miRNAs and downregulation of their target mRNAs, or downregulated miRNAs and upregulation of their mRNA targets. Thus, the list of putative targets of miRNAs was compared with tumor sample datasets. The list of target genes with anti-correlated profiles was subjected to Kegg Orthology (KO) analysis using the Database for Annotation, Visualization and Integrated Discovery (DAVID) webtool (http://david.abcc.ncifcrf.gov/). Using gene set enrichment analysis (GSEA), we also verified the existence of enriched gene signatures of cancer-related biological processes and signaling pathways, among target genes, for each thyroid tumor type.

### Download of gene expression data from *The Cancer Genome Atlas*

Gene expression data from 388 PTC samples was downloaded from cBioPortal for Cancer Genomics (www.cbioportal.org) and classified as low or high risk, according to clinical attributes related with aggressiveness, such as extra-thyroidal extension and presence of histologic lymphnode metastasis [19]. Samples were tested for normality using Kolmogrov-Smirnov test. Comparison between two groups was performed using Student’s t-test when samples presented Gaussian distribution, and non-parametric Mann-Whitney test when samples were considered not Gaussian. Samples were considered statistically different when p value<0.05. Overall survival curves were generated through cBioPortal webtool. Differential expression of target genes was automatically calculated by cBioPortal platform using Z-score ±2.0.

## Results

**Fig 1:**
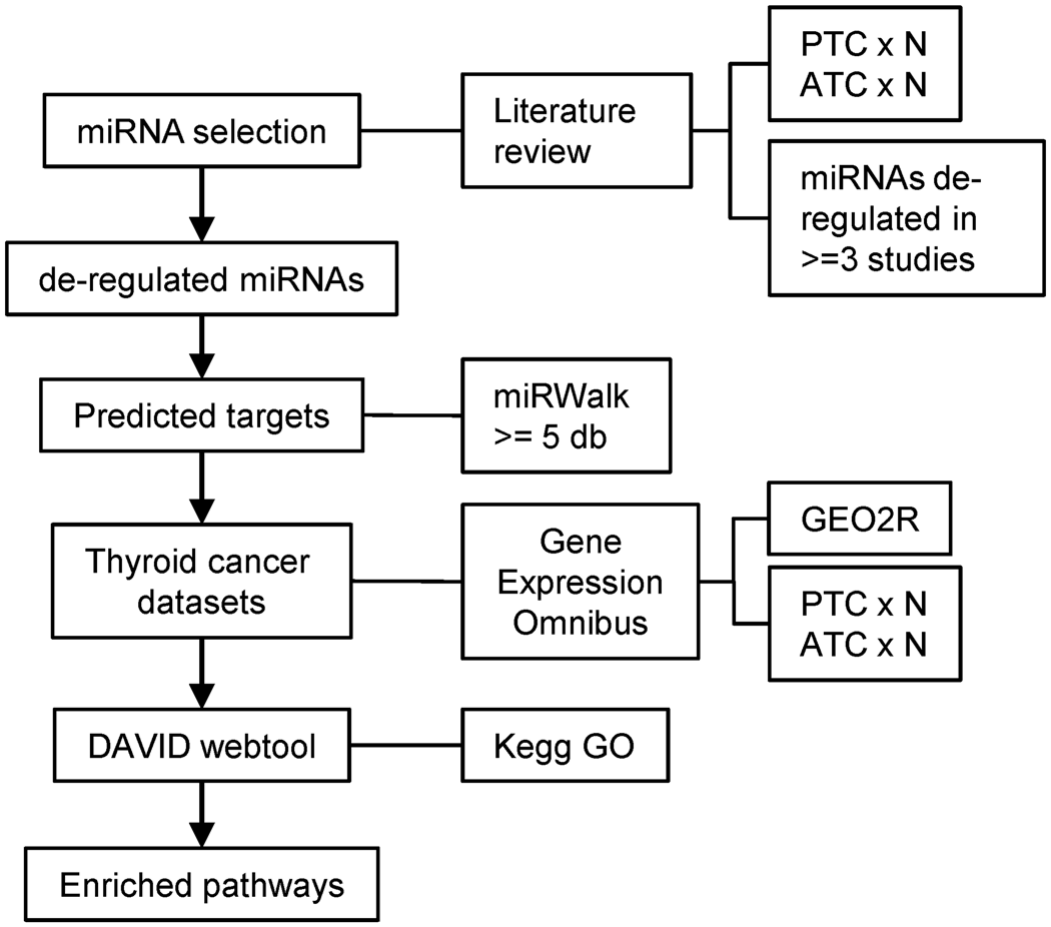
Strategy adopted for the construction of regulatory network of post-transcriptional regulation in thyroid cancer.

Several studies, using different platforms (microarray, qPCR Array and Next Generation Sequencing) and sources of nucleic acid (fresh, frozen, FFPE, and FNAB), have shown miRNA deregulation in different subtypes of thyroid cancer. We hypothesized that comparison of the predicted targets of miRNAs deregulated in PTC and ATC, with mRNA gene expression in thyroid cancer datasets, could provide relevant information regarding networks of post-transcriptional regulation (Fig 1). Firstly, to identify the miRNAs most described as altered in follicular cell-derived PTC and ATC thyroid tumors, we searched for the terms “miRNA” and “thyroid cancer” to select research articles that compared miRNA gene expression profiles between normal and human tumor samples (S1 Fig). As shown in S1 Table, a total of 15 studies, matching the criteria described in Material and Methods section, were included in the analysis, including 11 for PTC and 4 for ATC. One non-English study and 18 reviews were excluded.

Seventeen miRNAs were identified as deregulated in both subtypes of thyroid tumors by at least one study, despite their histological features. Particularly, the miRNAs *miR-221/222* were consistently described as overexpressed in at least three studies in both histological subtypes, suggesting a crucial role for these miRNAs in thyroid tumorigenesis. A total of 69 unique mature miRNAs were described as differentially expressed in PTC. Although twelve miRNAs were described as deregulated by three or more studies, two of them showed inconsistent expression patterns between studies and were excluded. We then selected the remaining set of 10 miRNAs (*miR-146b-5p*, *miR-221-3p*, *miR-222-3p*, *miR-181b-5p*, *miR-155-5p*, *miR-34a-5p*, *miR-138-5p*, *miR-187-3p*, *miR-224-5p* and *miR-31-5p*) reported as deregulated in three or more studies, with concordant expression patterns in (Table 1). In ATC, we selected the upregulated miRNAs, *miR-221/222*, and the downregulated miRNAs *let-7c*, *miR-125b-5p*, *miR-26a-5p*, *miR-30a-5p* and *miR-30d*, which had concordant expression in at least 3 studies. The complete list of differentially expressed miRNAs in thyroid cancer is shown in S2 Table.

**Table 1:** Differentially expressed miRNAs in PTC and ATC. This table represents the list of miRNAs described as deregulated in thyroid cancer samples by at least 2 different studies. MiRNAs selected for regulatory network construction are shown in bold italic.

	PTC											ATC			
miRNA ID	Jacques	Huang Y	Lassalle	Nikiforova	Agretti	Sheu	Pallante	He	Schwertheim	Yip	Swierniak	Braun	Visone	Nikiforova	Schwertheim
***miR-221-3p***		***up***	***up***	***up***	***up***	***up***	***up***	***up***	***up***	***up***		***up***		***up***	***up***
***miR-146b-5p***		***up***	***up***	***up***	***up***	***up***		***up***	***up***	***up***	***up***				***up***
***miR-222-3p***		***up***	***up***	***up***	***up***	***up***	***up***		***up***	***up***		***up***	***up***	***up***	***up***
***miR-181b-5p***			***up***	***up***		***up***	***up***		***up***					***up***	
***miR-155-5p***				***up***	***up***			***up***		***up***				***up***	
***miR-34a-5p***	***up***	***up***	***up***					***up***							
***miR-26a-5p***			down					down	up			***down***	***down***		***down***
***miR-224-5p***				***up***	***up***		***up***						***down***	***up***	
***miR-138-5p***								***down***		***down***	***down***	up			
***miR-187-3p***				***up***	***up***						***up***			up	
***miR-31-5p***			***up***	***up***						***up***					
***miR-125b-5p***							up		up			***down***	***down***		***down***
***let-7c***												***down***	***down***		***down***
***miR-30a-5p***												***down***	***down***		***down***
***miR-30d***												***down***	***down***		***down***
*miR-29b-3p*			up					up				down	down		
*miR-29c-3p*	up							up				down			
*miR-181a-5p*							up	up				down			
*miR-203a*			up								up		down		
*miR-21-5p*			up						up			up			up
*miR-100-5p*			down								down	down			
*miR-130a-3p*			down								down	down			
*miR-15b-5p*			down				down					down			
*miR-15a-5p*		up	up												
*miR-199a-5p*			down				down					down			
*miR-144-5p*		down									down				
*miR-7-5p*		down	down									down			
*miR-34b-5p*	up	up								down					
*miR-125a-5p*												down	down		
*miR-138-3p*												down	down		
*miR-145-5p*												down	down		
*miR-151a-5p*												down	down		
*miR-99a-5p*												down	down		
*miR-99b-5p*												down	down		
*let-7a-5p*												down	up		
*let-7f-5p*												down	up		
*miR-181c-5p*							up	up							
*miR-183-5p*	up										up				
*miR-213*							up	up							
*miR-21-3p*								up			up				
*miR-220*							up	up							
*miR-221-5p*		up									up				
*miR-222-5p*								up			up				
*miR-345-5p*			down					down							
*miR-34a-3p*	up	up													
*miR-451a*			down								down				
*miR-551b-3p*		up									up				
*miR-7-2-3p*		down									down				
*let-7d-5p*											down	down			
*miR-130b-3p*										down		up			
*miR-195-5p*											down	down			
*miR-204-5p*											down	down			
*miR-486-5p*											down	down			

To understand the role of post-transcriptional regulation exerted by the miRNAs deregulated in thyroid cancer, we used miRWalk program [20] to generate lists of the predicted targets of each differentially expressed miRNA. This program predicts miRNA:mRNA interactions using eight different algorithms, allowing the selection of miRNA targets that are simultaneously predicted by two or more algorithms. To increase reliability, however, we only selected targets predicted by five or more algorithms.

We next searched the GEO database for gene expression datasets of follicular cell-derived tumors, focusing on studies comparing gene expression between PTC or ATC and normal thyroid tissue. Based on the criteria described in the Materials and Methods section, we selected 5 studies that comprised the gene expression data of 203 samples, including PTC, ATC, and normal thyroid tissue (S3 Table). All five datasets comprised a total of 101 PTC samples and 69 normal thyroid tissues. Three of the selected datasets comprised ATC samples (20 tumors and 54 normal thyroid tissue samples).

**Fig 2:**
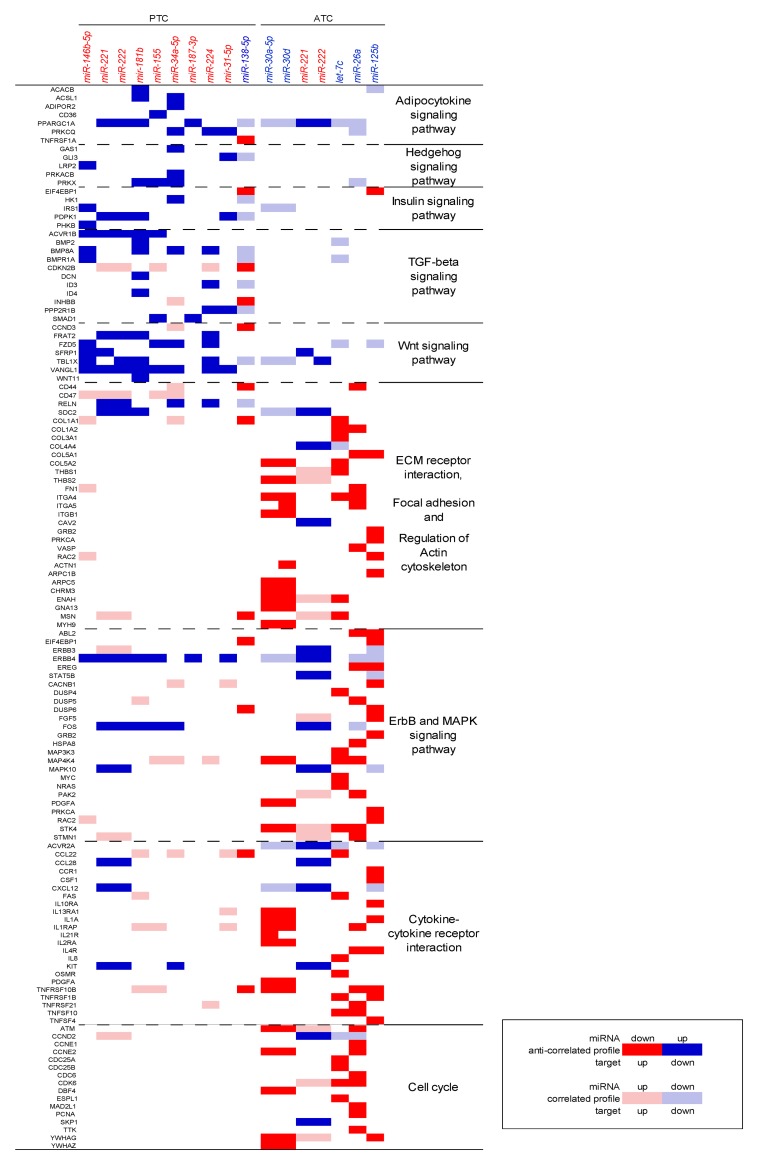
Enriched gene signatures identified by Gene Set Enrichment Analysis of miRNA targets.

Gene Set Enrichment Analysis (GSEA) of the differentially expressed genes in the PTC datasets, and DAVID webtool (david.abcc.ncifcrf.gov) analysis of the ATC datasets revealed, as expected, pathways commonly altered in thyroid cancer, including MAPK, TGF-beta, p53 signaling, and cell cycle (S4 Table). We then searched the datasets for miRNAs whose expression anti-correlated with their respective targets. We excluded predicted miRNA:mRNA interactions with concordant expression patterns (i.e., the miRNA and its respective target both up or downregulated). The resulting list of anti-correlated predicted miRNA:mRNA interactions was then analyzed by DAVID to identify enriched gene signatures. GSEA revealed that the selected miRNAs could regulate some of the commonly altered pathways in thyroid cancer, and also pathways not previously described as altered in this type of cancer. We observed the enrichment of TGF-beta, insulin, and adipocytokine pathways among the targets of miRNAs deregulated in PTC. In ATC specifically, the enriched pathways comprised cell cycle regulators, and the p53, MAPK, and ERBB pathways, as well as the regulation of migratory and invasive behavior, such as regulation of actin cytoskeleton and focal adhesion (Table 2 and Fig 2).

**Table 2:** Gene Set Enrichment Analysis.

Enriched pathways	Gene count	%	Fold	p-value
PTC	648			
*Adipocytokine signaling pathway*	10	1.5	3.79	*0*.*001071*
*Hedgehog signaling pathway*	8	1.2	3.63	*0*.*005758*
*TGF-beta signaling pathway*	11	1.7	3.21	*0*.*001937*
*Insulin signaling pathway*	11	1.7	2.07	*0*.*037866*
*Melanogenesis*	8	1.2	2.05	0.091789
*Wnt signaling pathway*	11	1.7	1.85	0.070756
ATC	441			
*ECM-receptor interaction*	13	2.40	2.15	*0*.*016442*
*p53 signaling pathway*	16	2.95	5.03	*3*.*32E-07*
*Focal adhesion*	24	4.43	2.55	*4*.*84E-05*
*Cell cycle*	18	3.32	3.08	*5*.*69E-05*
*Apoptosis*	12	2.21	2.95	*0*.*002166*
*ErbB signaling pathway*	12	2.21	2.95	*0*.*002166*
*Cytokine-cytokine receptor interaction*	23	4.24	1.88	*0*.*004847*
*MAPK signaling pathway*	23	4.24	1.84	*0*.*006063*
*Regulation of actin cytoskeleton*	18	3.32	1.79	*0*.*021906*

**Fig 3:**
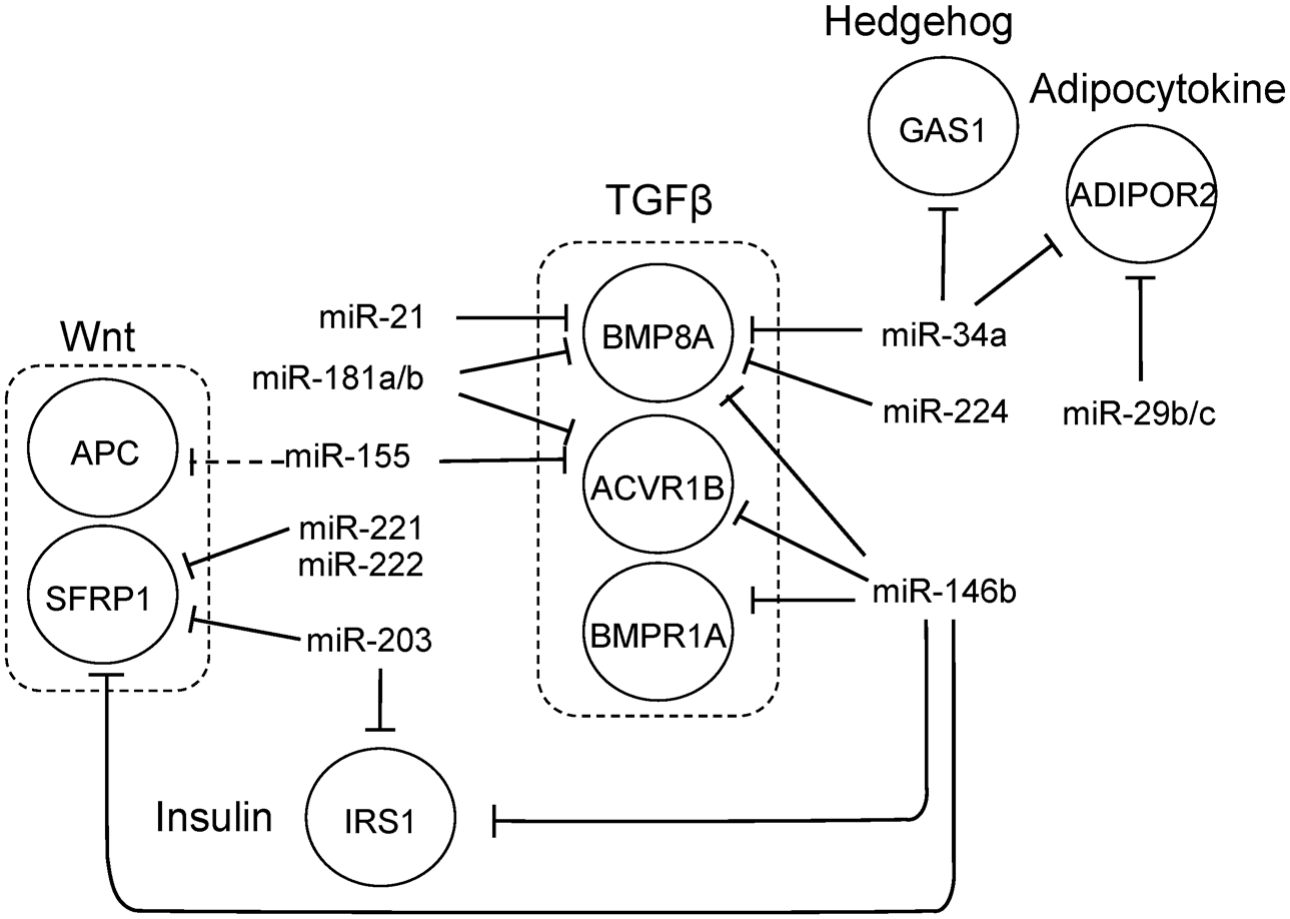
Example of regulatory networks generated by Gene Set Enrichment Analysis in PTC.

Our analysis showed that the TGF-beta pathway members *ACVR1B*, *BMPR1A* and *BMP8A* were potentially regulated by *miR-146b-5p*, *miR-221/222*, *miR-181a/b* and *miR-155* in PTC (Fig 3). Although our analysis didn’t show statistically significant enrichment for the Wnt signaling pathway, we did observe that *miR-146b-5p* and *miR-221* could regulate *SFRP1*, an inhibitor of this pathway. Our analysis also showed that *miR-34a-5p* could regulate *GAS1*, an inhibitor of the Hedgehog pathway, and *ADIPOR2*, which encodes the adiponectin receptor, in the PTC datasets. MiRNAs *miR-224* and *miR-31* could target *PRKCQ* and *miR-146b-5p* could target *IRS1*, which encodes the insulin receptor substrate-1. Additionally, *CCND3* (cyclin D3) could be modulated by *miR-138* in the PTC datasets.

**Fig 4:**
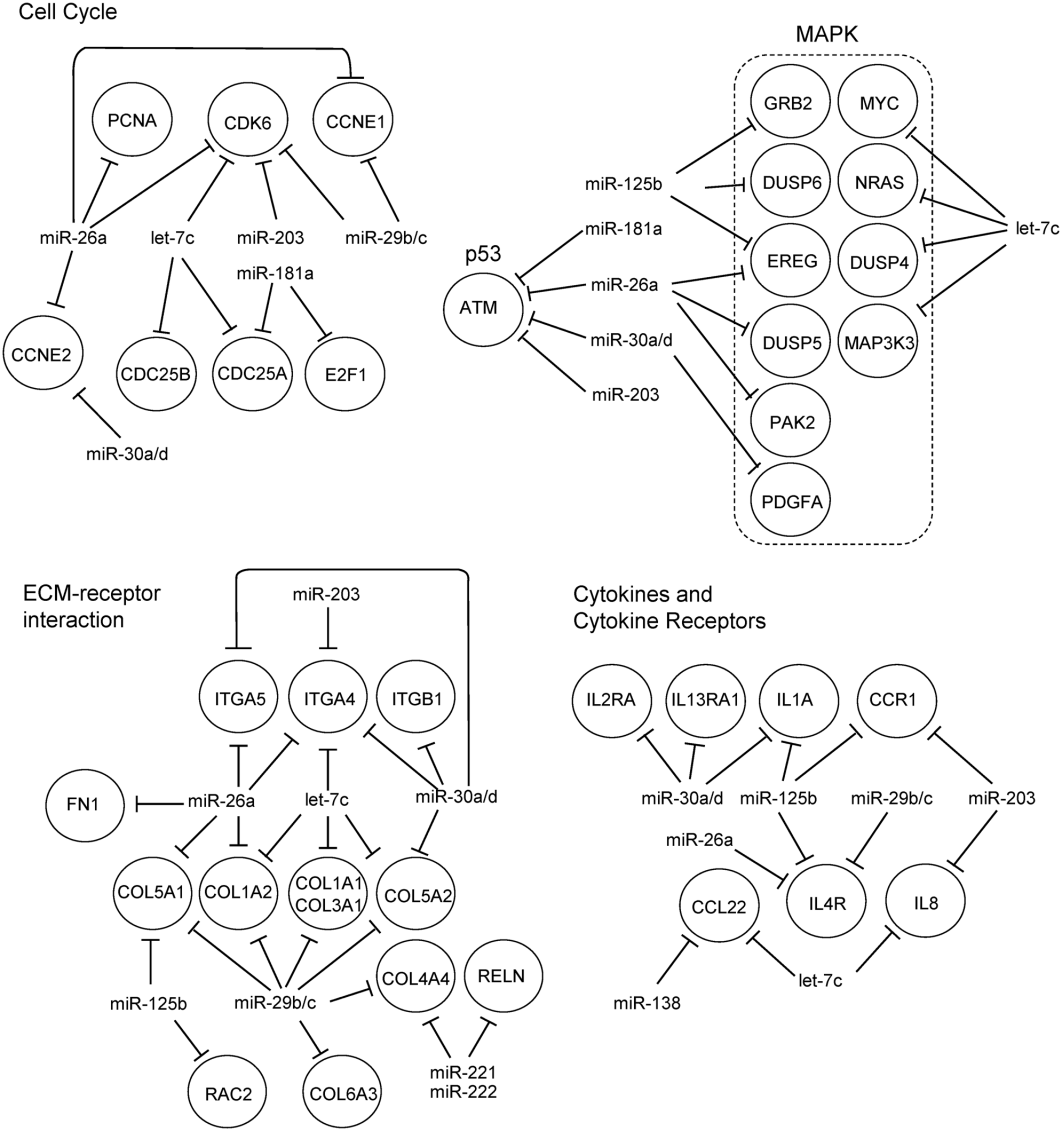
Example of regulatory networks generated by Gene Set Enrichment Analysis in ATC.

In the ATC datasets, our analysis suggested that miRNAs *miR-30a/d* and *miR-26a* could target *ATM* and cyclins E1 and 2 (*CCNE1* and *CCNE2*), thus contributing to modulation of the p53 pathway and cell cycle control, respectively (Fig 4). Cell cycling could also be regulated by miRNAs *let-7c* and *miR-26a* by repressing *CDK6*, *CDC25A/B* and *PCNA*. These miRNAs, along with *miR-125b* and *let-7c*, could also modulate genes involved in extracellular matrix receptor interactions and the actin cytoskeleton, including integrins α4, α5 and β1, and collagens 1A1, 1A2, 3A1, 5A1 and 5A2. Additionally, *RAC2* and *FN1* could be modulated by *miR-125b* and *miR-26a*, respectively. Notably, our analysis also suggested regulation of cytokines and their receptors in ATC. In particular, miRNAs *miR-125b*, *miR-30a/d*, *miR-26a*, and *let-7c* could cooperate to regulate interleukins 1 alpha (*IL1A*) and 8 (*IL8*), as well as the interleukin receptors *IL4R*, *IL13RA1*, *IL2RA* and *CCR1*, found overexpressed in ATC datasets.

Furthermore, we could not ignore the possibility that other miRNAs differentially expressed in thyroid cancer excluded from the analysis could also influence the regulation of these signaling pathways. Although the miRNAs *miR-203a*, *-29b/c* and *181a* did not match the criteria for miRNA selection, we did observe an interesting expression pattern for these miRNAs, as they were upregulated in PTC and downregulated in ATC. The GSEA of the potential targets of these miRNAs revealed that these miRNAs target genes involved in the same altered biological processes and pathways (data not shown). Upregulated miRNAs in PTC could regulate members of the TGF-beta, Hedgehog, Wnt, and insulin signaling pathways. In ATC, decreased expression of these miRNAs could explain modulation of p53 signaling, cell cycle, cytokine-cytokine receptor interaction, and ECM-receptor interaction. Additionally, *miR-21* and *miR-15a* and b, deregulated in several types of cancer, could also modulate these pathways in PTC and ATC.

**Fig 5:**
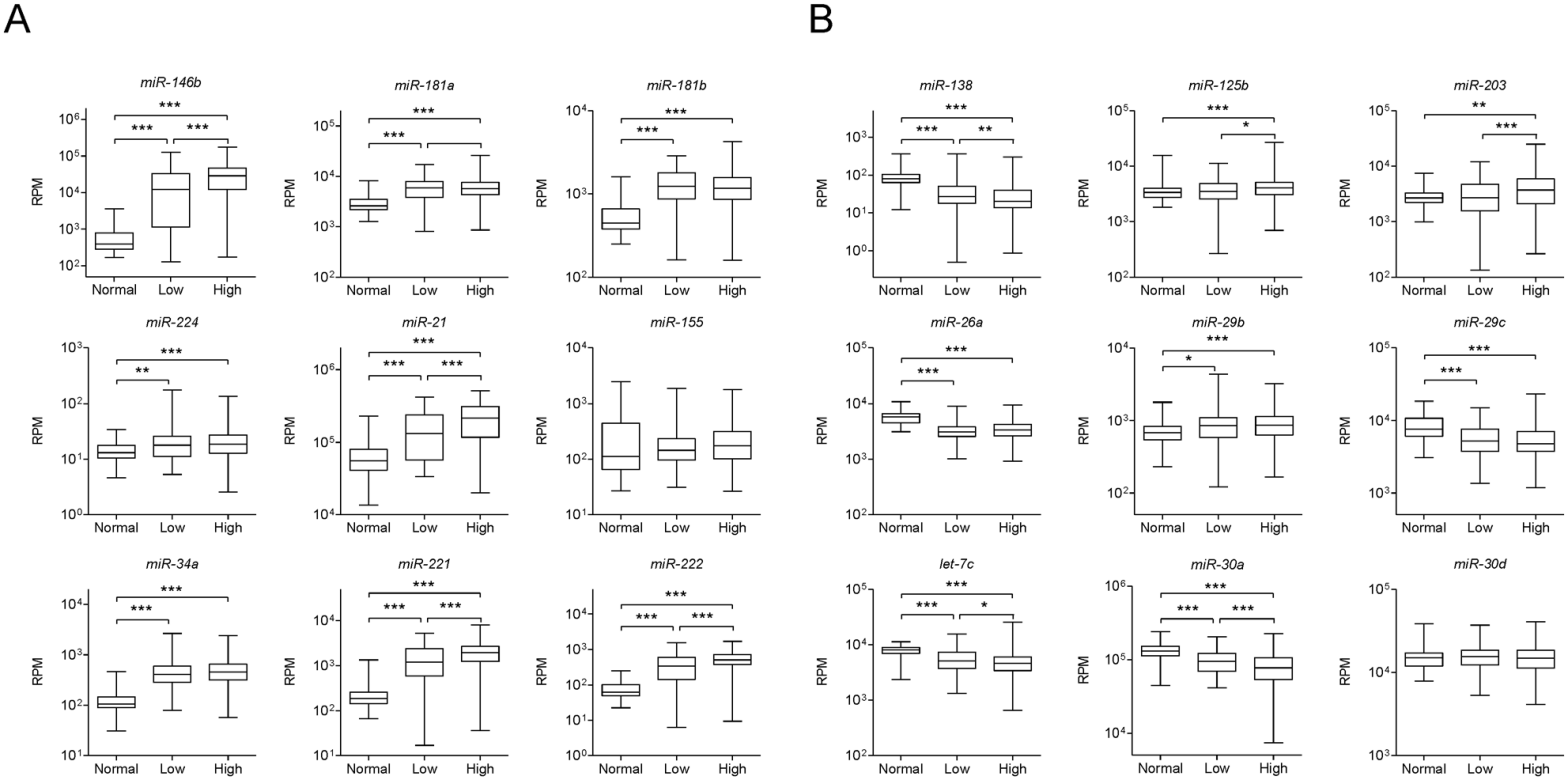
Differential expression of miRNAs frequently described as deregulated in ATC correlates with increased risk in PTC. MiRNA expression information of the miRNAs frequently described as deregulated in (A) PTC and (B) ATC was downloaded from cBioPortal for Cancer Genomics (www.cbioportal.org). Non-parametric Mann-Whitney test was performed comparison between groups. * p-value<0.05, ** p-value<0.01, *** p-value<0.001.

Using the cBioPortal for Cancer Genomics [21], we assessed the genomic, transcriptomic and clinical data from a total of 388 PTC patients from The Cancer Genome Atlas (TCGA) Research Network (http://cancergenome.nih.gov/). We observe that the expression of many of the miRNAs described as deregulated in ATC is also observed in PTC samples and correlates with increased risk (Fig 5).

**Fig 6:**
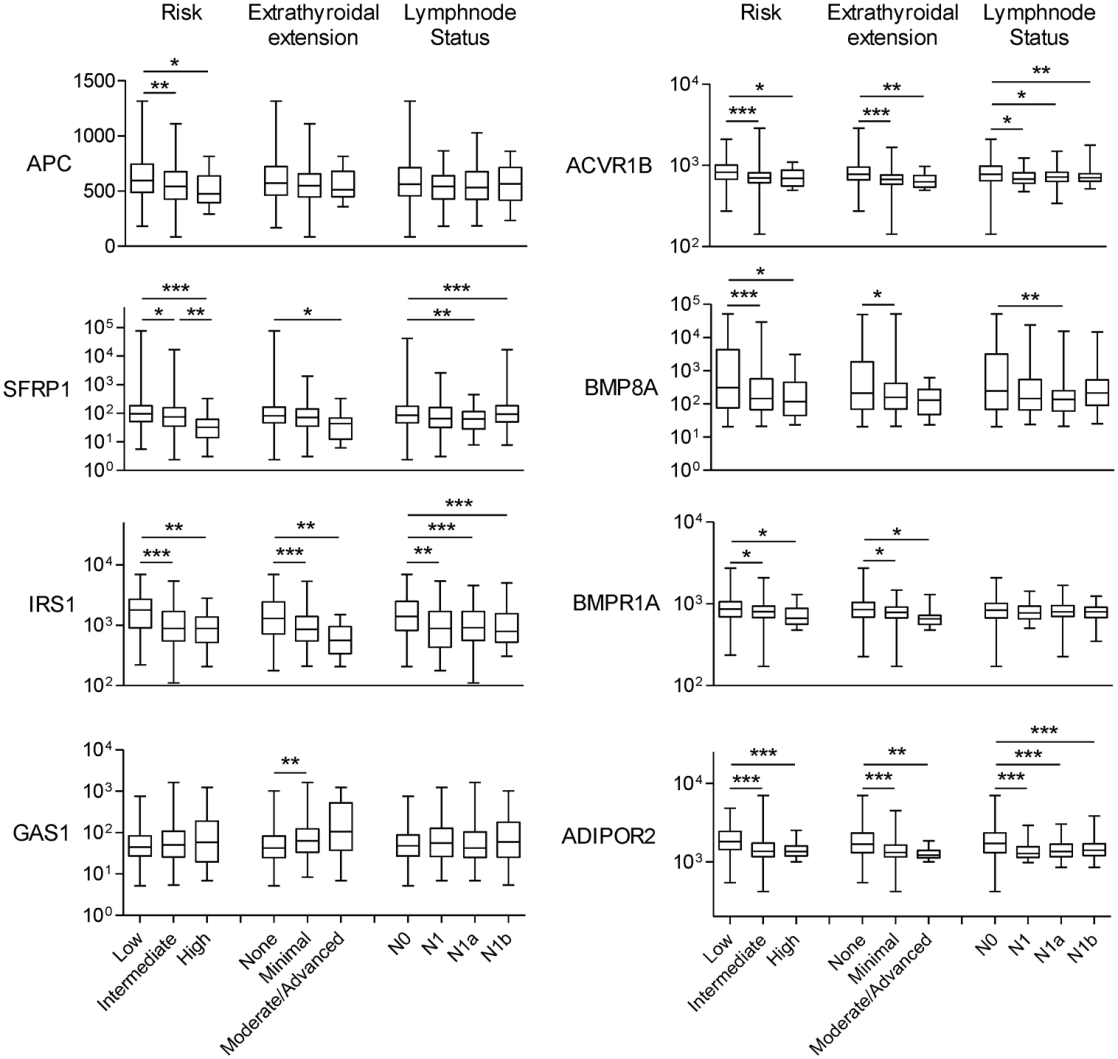
Abnormal expression of target genes in PTC is associated with increased risk, extra-thyroidal extension and lymphnode metastasis in PTC. Non-parametric Mann-Whitney test was performed comparison between groups. * p-value<0.05, ** p-value<0.01, *** p-value<0.001.

**Fig 7:**
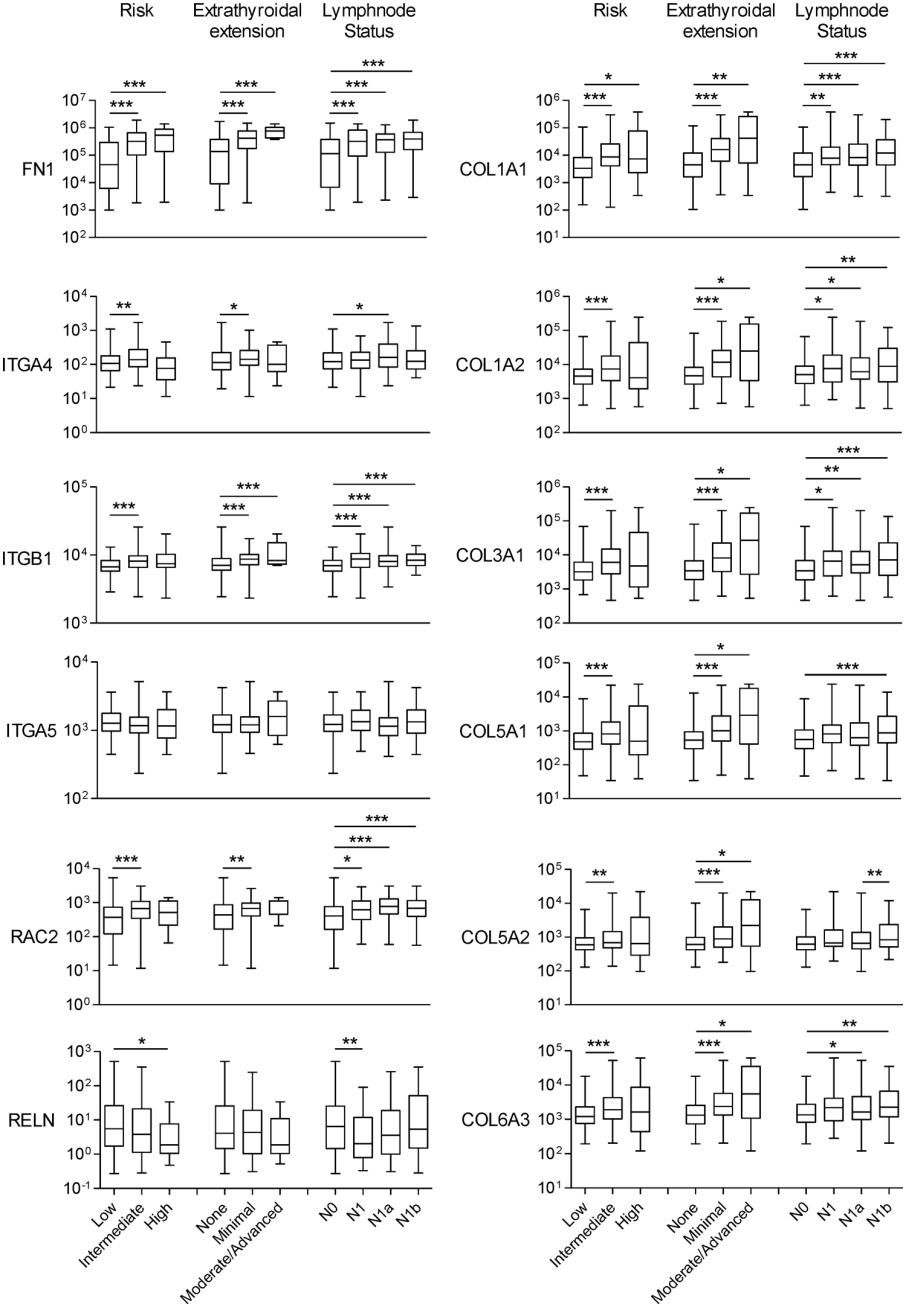
Cytokines and cytokine receptors deregulation is associated with increased risk, extra-thyroidal extension and lymphnode metastasis in PTC. Non-parametric Mann-Whitney test was performed comparison between groups. * p-value<0.05, ** p-value<0.01, *** p-value<0.001.

**Fig 8:**
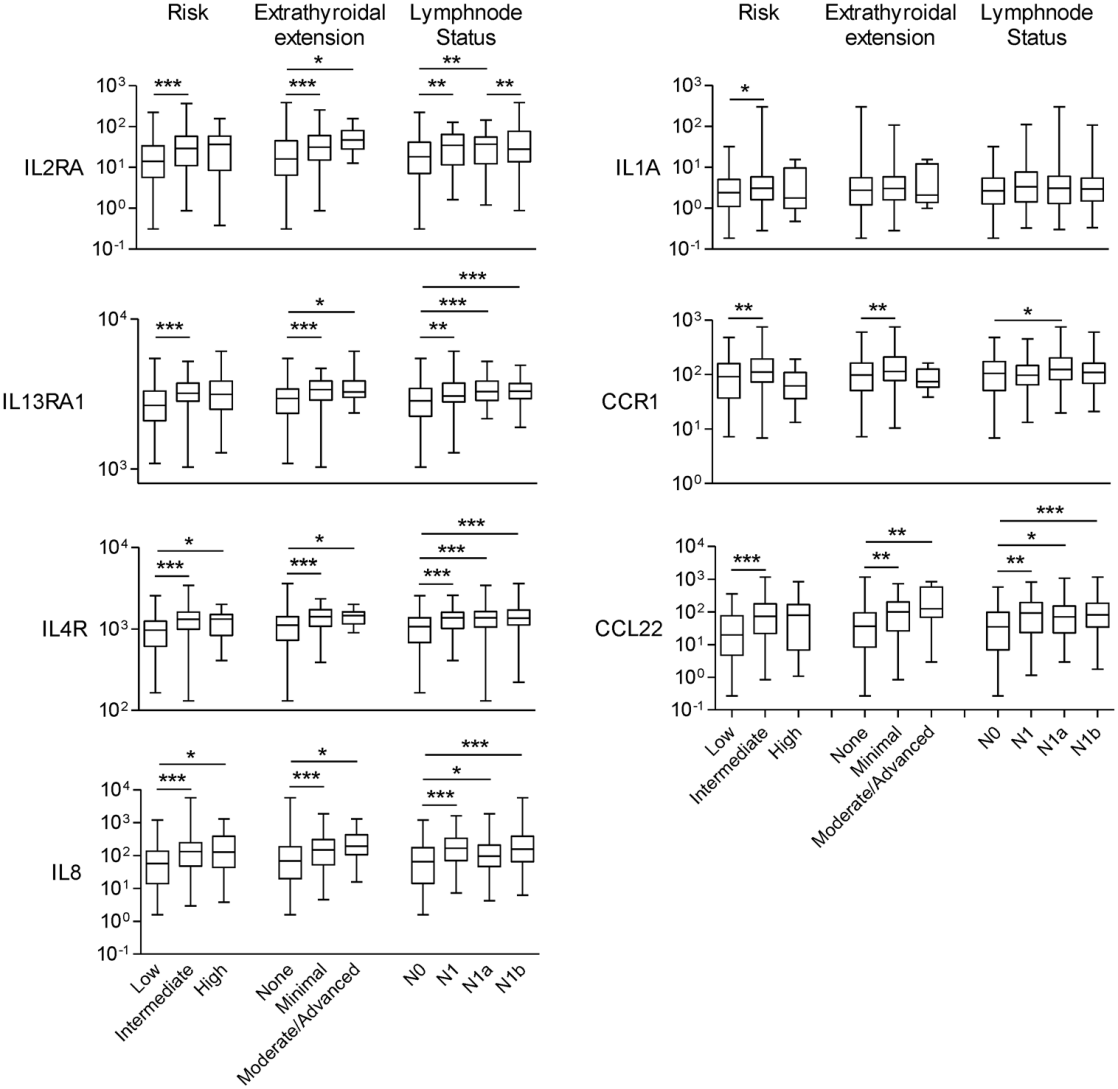
ECM remodeling molecules deregulation is associated with increased risk, extra-thyroidal extension and lymphnode metastasis in PTC. Gene expression information was downloaded from cBioPortal for Cancer Genomics (www.cbioportal.org). Non-parametric Mann-Whitney test was performed comparison between groups. * p-value<0.05, ** p-value<0.01, *** p-value<0.001.

**Fig 9:**
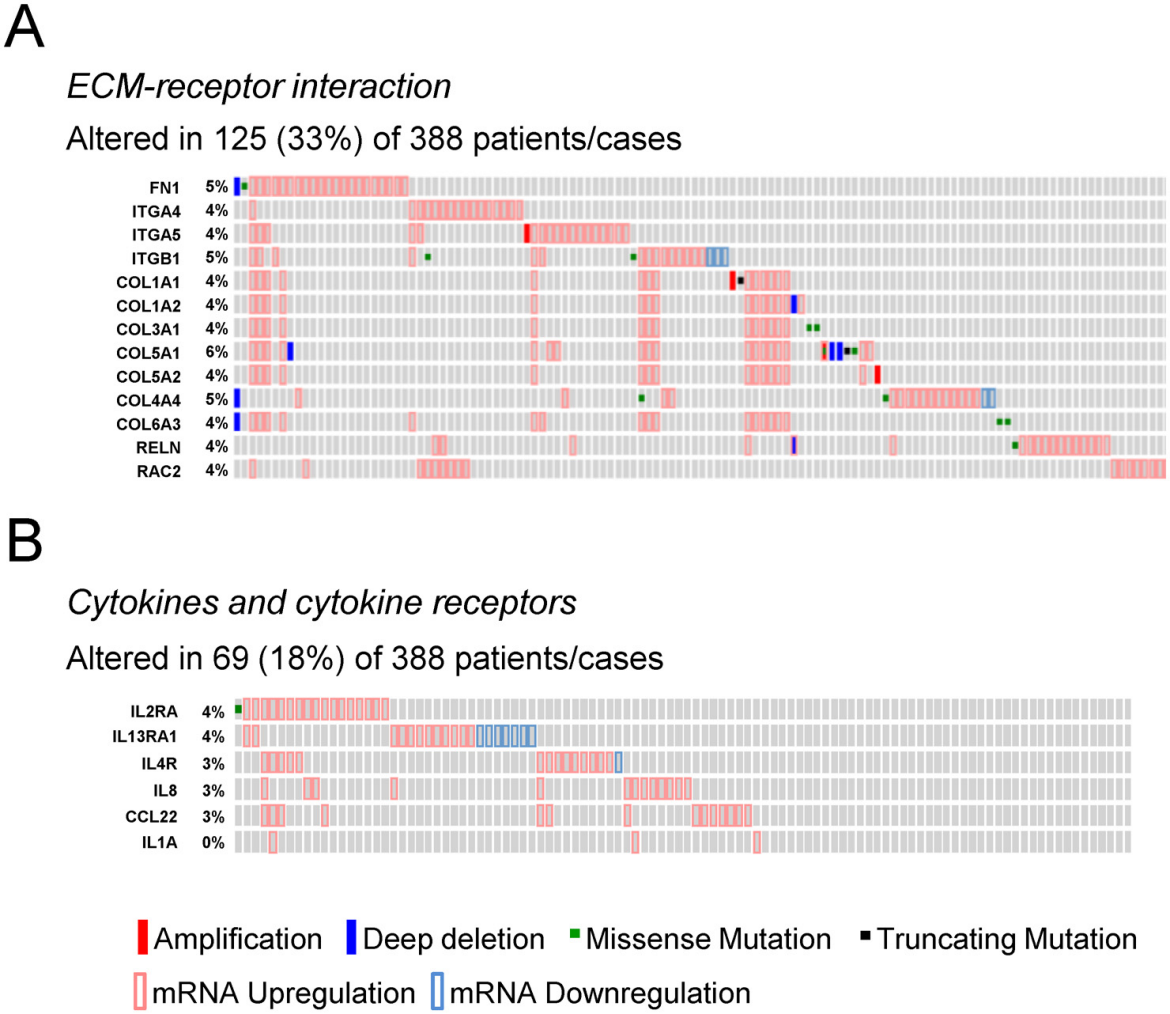
Abnormal gene expression of ECM remodeling molecules and cytokines and cytokine receptors is frequent in PTC. Genetic alteration information was obtained through the cBioPortal for Cancer Genomics. The list of target genes enriched in (A) *ECM-receptor interaction* and (B) *Cytokines and cytokine receptors* ontology classes in ATC was submitted to cBioPortal.

**Fig 10:**
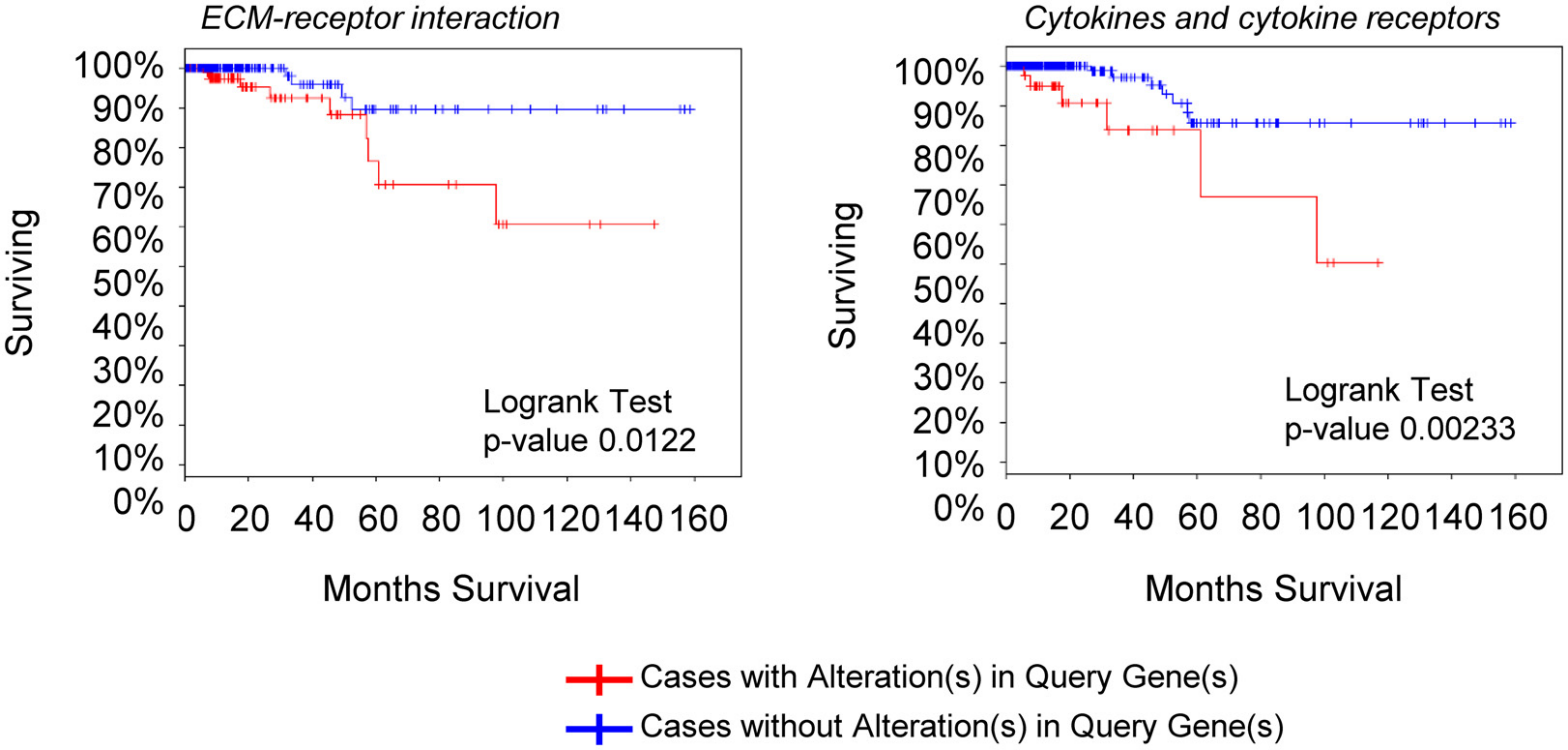
Alteration in ECM remodeling molecules and cytokines and cytokine receptors is associated with decreased survival rate in PTC. Clinical data was assessed through cBioPortal for Cancer Genomics. The list of target genes enriched in *ECM-receptor interaction* and *Cytokines and cytokine receptors* ontology classes in ATC was submitted to cBioPortal.

We then analyzed the gene expression pattern of the enriched miRNA target genes used to construct the regulatory networks shown in Figs 3 and 4. In PTC, the differential expression of TGF-β, Wnt, insulin pathway members and the adipocytokine receptor 2 is significantly correlated with increased risk, extra-thyroidal extension and presence of lymphnode metastasis (Fig 6). Interestingly, although the enrichment of ECM remodeling molecules and cytokines and cytokine receptors was observed only in ATC datasets, the dysregulation of these genes in PTC samples is associated with aggressive phenotype (Figs 7 and 8). The alterations found in these genes are predominantly at gene expression level and is observed in 33% (125 of 388) and 18% (69 of 388) of the cases, for ECM remodeling genes and cytokine and cytokine receptor gene, respectively (Fig 9). When comparing patients with and without abnormal expression of these genes, the group of patients with aberrant gene expression of at least one of the ECM remodeling and cytokine genes presents diminished overall survival rate in comparison with those without alterations in these genes (Fig 10). When we considered all the list of targets identified by the GSEA, gene expression abnormality in *ECM-receptor interaction* and *Cytokines-cytokine receptors* ontology classes is present in 60% and 46% of the samples, respectively, however without statistical significance in overall survival curves (data not shown). Any statistical significance could be observed in overall survival curves regarding differential expression of genes enriched in *Wnt*, *TGF-β*, *Hedgehog*, *cell cycle* and *MAPK* ontology classes in PTC (data not shown).

## Discussion

Due to the modulation of key tumor suppressors and oncogenes, miRNAs have emerged as a new hallmark of cancer, contributing both to its development and progression. According to the *clinicaltrials*.*gov* database, pre-clinical and clinical studies have started to unveil the therapeutic potential of miRNAs (https://clinicaltrials.gov). However, due to the high number of possible miRNA/mRNA interactions, the overall panorama of post-transcriptional gene regulation in thyroid cancer remains poorly understood, especially regarding aggressive, undifferentiated thyroid cancer. Most investigations of the biological role of miRNAs in thyroid cancer are based on “cherry picking” targets from lists generated by computational algorithms, such as TargetScan, PicTar and miRANDA. In the present study, we aligned miRNA target prediction, publicly available gene expression datasets and Gene Set Enrichment Analysis (GSEA), to identify the most affected pathways and biological processes by the miRNAs most frequently described as deregulated in differentiated and undifferentiated thyroid cancer. The use of microarray datasets allowed us to construct robust networks of gene expression, which have proven useful for assessing gene expression data from rare, non-operable types of thyroid cancer, such as ATC. In total, global gene expression was assessed in approximately one hundred PTC and 20 ATC samples, and compared with more than 60 normal thyroid samples.

**Fig 11:**
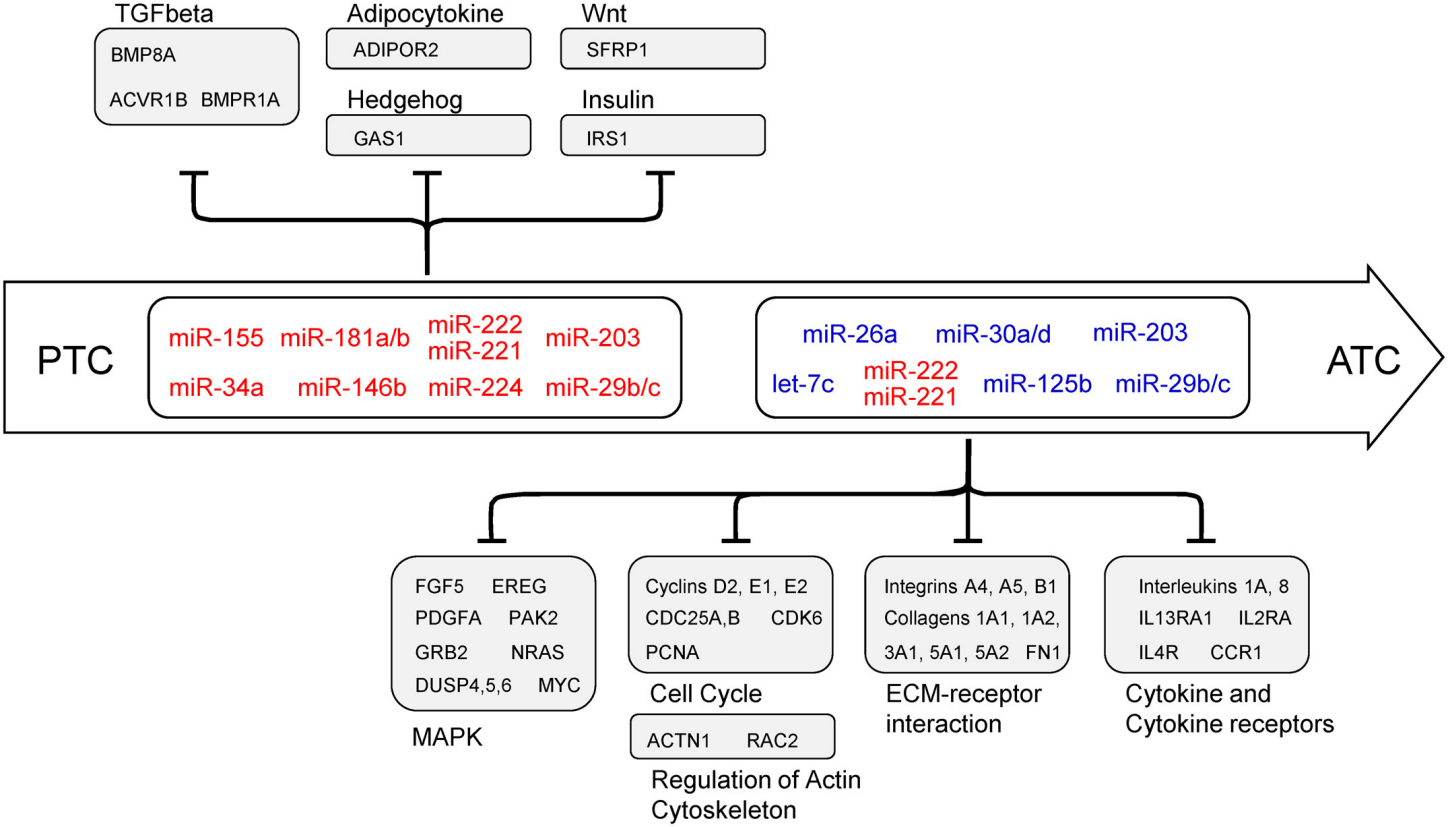
Post-transcriptional regulation by miRNAs in thyroid cancer. MiRNAs altered in PTC target genes that modulate biological processes frequently altered in well-differentiated tumors, such as control of epithelial proliferation and self-renewal. In ATC, enriched pathways are related with aggressive behavior, such as extra-cellular matrix receptor interaction, focal adhesion and regulation of actin cytoskeleton. Additionally, enrichment of pathways not previously described in thyroid cancer were observed in PTC, such as adipocytokine and insulin signaling pathway, and in ATC, such as cytokine and cytokine receptors.

As illustrated in Fig 11, we observed that miRNAs altered in PTC target different members of pathways related to the regulation of epithelial cell proliferation and self-renewal, such as the TGF-β, Wnt, and Hedgehog pathways. Along with the highly expressed *miR-146b-5p*, the miRNAs *miR-221/222*, *miR-181a/b* and *miR-155* may actually attenuate TGF-β signal transduction by repressing not only the SMAD intracellular signaling components, but also activin receptor 1B (ACVR1B) and bone morphogenetic factor 8A (BMP8A), and contribute with tumor progression. Moreover, our analysis shows evidence that *miR-34a-5p* overexpression could explain the lower levels of ADIPOR2 in PTC, contributing with an aggressive behavior in PTC, as extra-thyroidal invasion, multicentricity, and higher TNM [22, 23].

On the other hand, aberrant expression of miRNAs may contribute to ATC’s aggressive behavior through the modulation of tumor-host interaction pathways, such as adhesion molecules, extracellular matrix receptors, and immune system mediators. The analysis of large datasets allowed us to observe that abnormal expression of the miRNAs frequently described as deregulated in ATC also in aggressive PTCs. These miRNAs might modulate different components of ECM, focal adhesion and actin cytoskeleton, corroborating previous findings that show crucial roles of miRNAs in regulating migration and invasion of thyroid cancer cells [24–28]. In ATC, these miRNAs may also contribute to sustained MAPK signaling, through targeting *NRAS*, *MYC* and *EREG*, and with the loss of cell cycle control, through targeting cyclins *D3*, *E1*, and *E2*, *CDC25A* and *B*, and *CDK6*. MiRNAs have also been shown to be important in cell cycle control in PTC, through the repression of *CDKN2A*, *CCND2* and *CDK6*, by miRNAs *miR-221/222*, *miR-1* and *miR-191* respectively [25, 29, 30].

**Fig 12:**
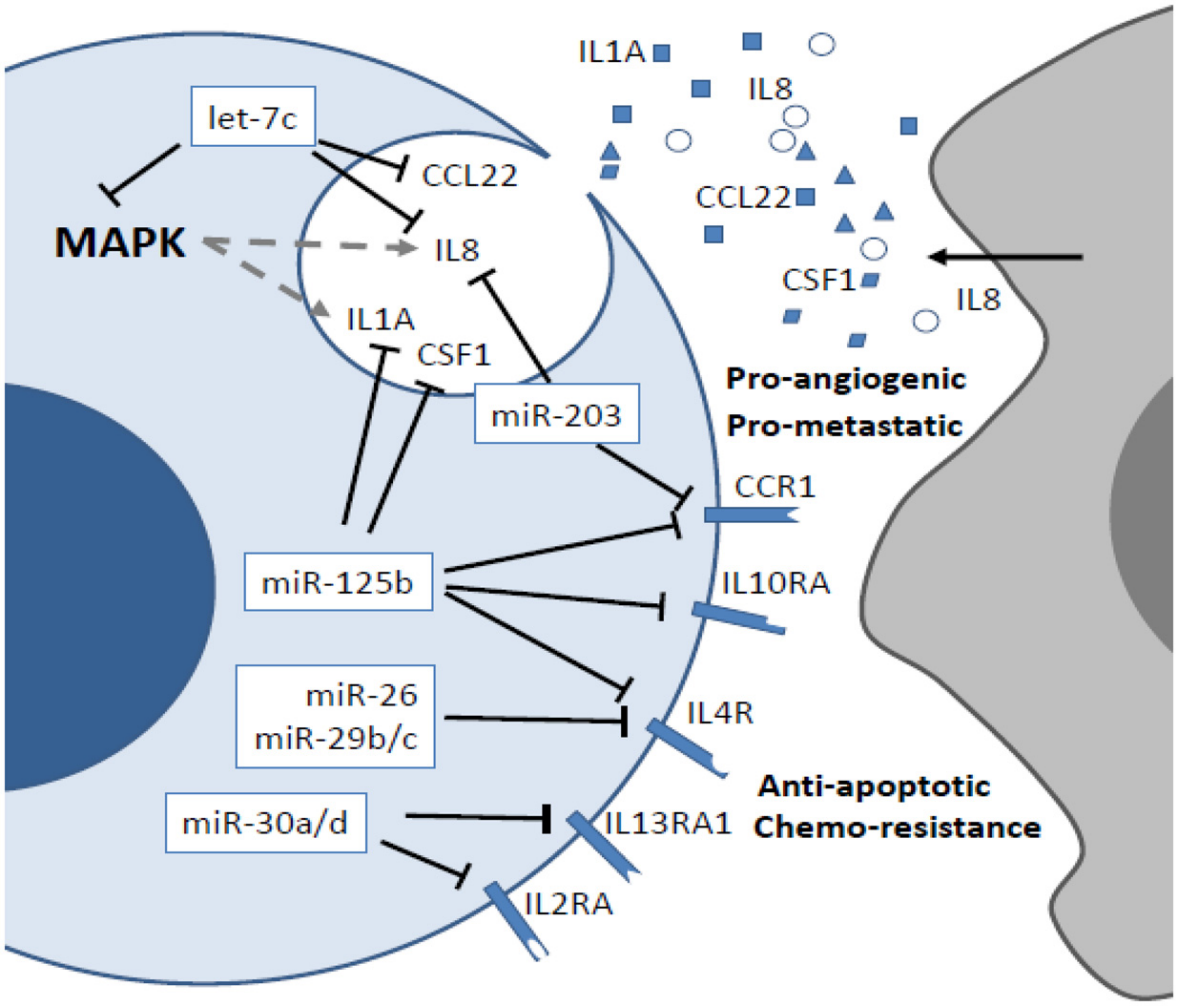
Post-transcriptional control of inflammatory responses in PTC and ATC. Tumor-associated macrophages (TAM) contribute with metastasis through secretion of IL8. *RET* activation induces secretion of IL1A and IL8. The decreased expression of miRNAs *miR-26*, *-29b/c*, *-30a/d*, *-125b*, *-203* and *let-7c* may lead to increased expression of interleukins IL1A, IL8, CCL22 and CSF1, which secretion generate pro-metastatic and –angiogenic responses in thyroid cancer cells. Increased expression of receptors IL2RA, IL4R, IL13RA1, IL10RA and CCR1 may favor chemo-resistance and survival by autocrine and paracrine stimulation.

Several studies have now shown the impact of the immune system on thyroid tumor growth and progression, with promising clinical implications [31]. Tumor-associated macrophages (TAMs) are frequently observed in thyroid tumors and contribute to tumor progression and metastatic spread through secretion of IL8 [32–34]. On the other hand, thyroid tumor cells may actively participate in leukocyte recruitment, as *RET* oncogenic activation induces upregulation of the pro-inflammatory interleukins IL1α and IL8 *in vitro* [35–37]. Additionally, interleukins 4 and 10, as well as their receptors, are involved in resistance to chemotherapy and Fas-induced apoptosis, in PTC cells [38–40]. Recent studies have also shown that increased risk of PTC associates with polymorphisms in different interleukins [41, 42]. However, the real contribution of miRNAs to the modulation of immune system mediators in thyroid cancer is not fully understood. Leone and colleagues demonstrated that *miR-1* post-transcriptionally regulates *CXCR4* in ATC [25]. Furthermore, the ATC-derived cell line FRO exhibits activation of the NFkB signaling pathway, which upregulates *miR-146a* to generate resistance to chemotherapeutic agents and survival [43]. In fact, NFkB pathway signaling is directly activated by a T1799A mutation in the *BRAF* oncogene, inducing the secretion of IL8 to increase tumor aggressiveness in thyroid cancer cells [37, 44]. As shown in Fig 12, our results suggest that the abnormal expression of several miRNAs could explain the aberrant expression of different interleukins and interleukin receptors in PTC and ATC, stimulating crosstalk between the tumor and the immune system, thus favoring leukocyte recruitment, tumor growth, and more aggressive behavior. Notably, our analysis also revealed potential post-transcriptional regulation of interleukins and interleukin receptors not previously reported in PTC and ATC. For example, *CCR1*, *IL1RAP*, *CCL22* and *CSF1* are all involved in metastatic dissemination, and their aberrant expression correlates with leukocyte infiltration and aggressive phenotype in various cancer types [45–48]. Importantly, gene expression data from The Cancer Genome Atlas (TCGA) [21] revealed that abnormal expression of ECM and cytokines genes is a frequent event in PTC (one third and one fifth of the cases, respectively) and is associated with aggressive behavior and decreased overall survival rate. This observation indicates that miRNA-mediated modulation of ECM and cytokine genes in PTC could contribute with progression to undifferentiated thyroid cancer.

In conclusion, by taking advantage of computational tools and gene expression data available in public depositories, we shed light on the panorama of post-transcriptional regulation exerted by miRNAs in thyroid cancer. Although infiltration of immune cells and the presence of inflammation may be helpful as prognostic markers, the modulation of immune system mediators as a therapeutic tool is not a reality for thyroid cancer. Our results indicate that the miRNAs most frequently described as deregulated in PTC and ATC may be involved in an intricate network, where a small number of miRNAs may act cooperatively to control different biological processes and orchestrate tumor progression and metastasis. Thus, the exogenous modulation of miRNAs that target interleukins within the tumor microenvironment could emerge as an alternative to suppress the protumorigenic interplay between tumor and host cells that favors tumor dissemination. The finding of miRNA:mRNA interactions not previously reported in PTC and ATC, particularly immune system mediators, may open the way to explore the potential of miRNAs as therapeutic tools for thyroid cancer.

## Supporting Information

S1 Fig Literature review for selection of miRNAs mostly described as deregulated in thyroid cancer. (TIF)

S1 Table miRNA profiling studies selected for analysis. (DOCX)

S2 Table miRNA de-regulation in PTC and ATC. (DOCX)

S3 Table Gene expression datasets selected for construction of regulatory network of post-transcriptional regulation. (DOCX)

S4 Table Gene Set Enrichment Analysis of thyroid cancer transcriptome. (DOCX)
