# Supplementary figures and images for: Integrated Analysis of Thyroid Cancer Public Datasets Reveals Role of Post-Transcriptional Regulation on Tumor Progression by Targeting of Immune System Mediators
Source: PLoS One. 2015 Nov 4;10(11):e0141726. doi: 10.1371/journal.pone.0141726 (PMC4633176; doi:10.1371/journal.pone.0141726)

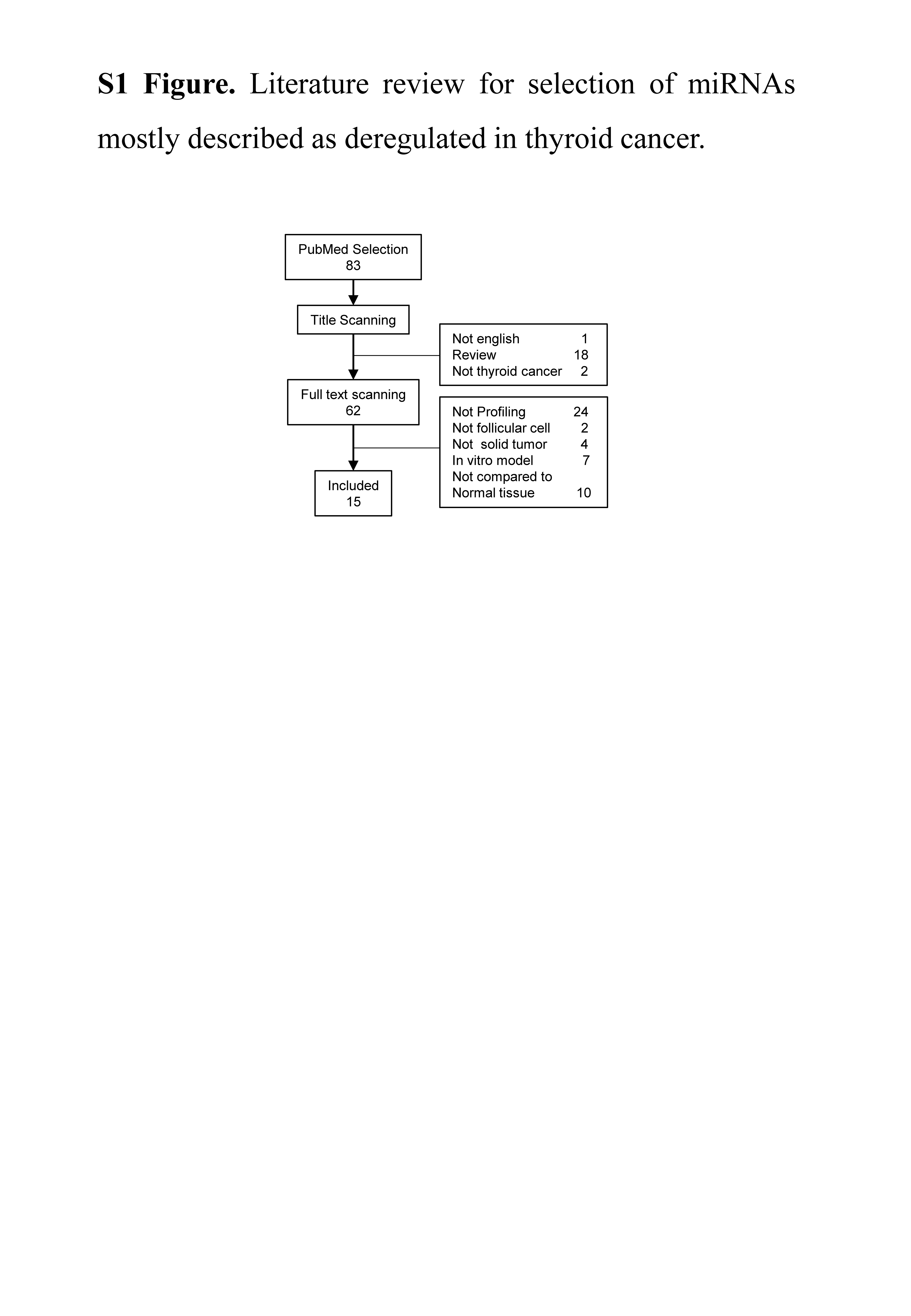

Supplement: S1 Fig — (TIF) [file pone.0141726.s001.tif]
