# Supplementary material for: Integrated Analysis of Thyroid Cancer Public Datasets Reveals Role of Post-Transcriptional Regulation on Tumor Progression by Targeting of Immune System Mediators
Source: PLoS One. 2015 Nov 4;10(11):e0141726. doi: 10.1371/journal.pone.0141726 (PMC4633176; doi:10.1371/journal.pone.0141726)
Supplement: S1 Table — (DOCX) [file pone.0141726.s002.docx]

**S1 Table.** miRNA profiling studies selected for analysis.

| Reference | Profiling Strategy | | | | Platform | |  | | Sample | |  |  |
| --- | --- | --- | --- | --- | --- | --- | --- | --- | --- | --- | --- | --- |
| PTC |  | |  | |  | |  | |  | |  |  |
| Jacques C et al.(46) | microarray | PTC x NT | | Agilent Technologies | | 866 human miRNAs | | frozen | | 25 FA, 38 OTT, 19 PTC, 10 TUMPs, and 53 NT. | | |
| Huang Y et al.(47) | microarray | PTC x NT | | Agilent Technologies | | 866 human miRNAs | | frozen | | 69 PTC and matched NT | | |
| Agretti P et al.(48) | set of seven miRNAs | PTC x NT, benign nodules x NT | | TaqMan miRNA Assay (Life) | |  | | FNA and frozen | | 43 PTC and 45 benign nodules (relative to NT) | | |
| Lassalle S et al.(49) | microarray | PTC x NT, FTC x NT | | non-commercial | | 1449 human miRNAs | | frozen | | 16 PTC (11 classical PTC, 5 fvPTC), 6 FTC, 7 FA and matching NT | | |
| Yip L et al. (50) | PCR Array | PTC x NT | | FlexmiR version 8 (Exiqon) | | 319 human miRNAs | | FFPE and frozen | | 12 PTC and 4 NT | | |
| Schwertheim S et al. (51) | two sets of five miRNAs | PTC x NT, ATC x NT | | TaqMan miRNA Assay (Life) | |  | | FFPE tissue | | 15 PDTC, 9 PTC , 9 ATC and 4 NT | | |
| Sheu SY et al. (52) | set of five miRNAs | PTC x NT | | TaqMan miRNA Assay (Life) | |  | | FFPE tissue | | 50 classical PTC, 71 tall cell PTC, 56 fvPTC and matched NT | | |
| Nikiforova MN et al. (14) | TaqMan array | PTC x NT, FTC x NT, ATC x NT | | TaqMan miRNA Panel (Life) | | 158 human miRNAs | | FNA and frozen | | 23 PTC (18 classical PTC, 5 fvPTC), 9 FTC, 8 FA, 4 ATC, 4 PDTC, 2 MTC, 5 hyperplastic nodules and 5 NT | | |
| Pallante P et al. (10) | microarray | PTC x NT | | non-commercial | | 245 human and mouse miRNA genes | | FNA and frozen | | 30 PTC and 10 NT | | |
| He H et al. (9) | microarray | PTC x NT | | non-commercial | | 235 Human miRNAs | | frozen | | 20 PTC and matched NT | | |
| Swierniak M et al. (53) | Next Generation Sequence | PTC x NT | | SOLiD (Life) | |  | | frozen | | 14 PTC, unaffected adjacent tissue and 14 NT | | |
| ATC |  |  | |  | |  | |  | |  | | |
| Braun J et al.(21) | microarray | ATC x NT | | not available | | not available | | not available | | 3 ATC and 3 NT | | |
| Schwertheim S et al.(51) | two sets of five miRNAs | PTC x NT, ATC x NT | | TaqMan miRNA Assay (Life) | |  | | FFPE | | 15 PDTC, 9 PTC , 9 ATC and 4 NT | | |
| Nikiforova MN et al. (14) | TaqMan array | PTC x NT, FTC x NT, ATC x NT | | TaqMan miRNA Panel (Life) | | 158 human miRNAs | | FNA and frozen | | 23 PTC (18 classical PTC, 5 fvPTC), 9 FTC, 8 FA, 4 ATC, 4 PDTC, 2 MTC, 5 hyperplastic nodules and 5 NT | | |
| Visone R et al. (12) | microarray | ATC x NT | | non-commercial | | not available | | not available | | 10 ATC and 10 NT | | |

|  |  |  |  |  |
| --- | --- | --- | --- | --- |
|  |  |  |  |  |
