# Supplementary material for: Integrated Analysis of Thyroid Cancer Public Datasets Reveals Role of Post-Transcriptional Regulation on Tumor Progression by Targeting of Immune System Mediators
Source: PLoS One. 2015 Nov 4;10(11):e0141726. doi: 10.1371/journal.pone.0141726 (PMC4633176; doi:10.1371/journal.pone.0141726)
Supplement: S2 Table — (DOCX) [file pone.0141726.s003.docx]

**S2 Table.** miRNA de-regulation in PTC and ATC.

|  | PTC | | | | | | | | | | |  | ATC | | | |
| --- | --- | --- | --- | --- | --- | --- | --- | --- | --- | --- | --- | --- | --- | --- | --- | --- |
| miRNA ID | Jacques | Huang Y | Lassalle | Nikiforova | Agretti | Sheu | Pallante | He | Schwertheim | Yip | Swierniak |  | Braun | Visone | Nikiforova | Schwertheim |
| ***`=*** |  | ***up*** | ***up*** | ***up*** | ***up*** | ***up*** | ***up*** | ***up*** | ***up*** | ***up*** |  |  | ***up*** |  | ***up*** | ***up*** |
| ***miR-222-3p*** |  | ***up*** | ***up*** | ***up*** | ***up*** | ***up*** | ***up*** |  | ***up*** | ***up*** |  |  | ***up*** | ***up*** | ***up*** | ***up*** |
| ***miR-146b-5p*** |  | ***up*** | ***up*** | ***up*** | ***up*** | ***up*** |  | ***up*** | ***up*** | ***up*** | ***up*** |  |  |  |  | up |
| ***miR-181b-5p*** |  |  | ***up*** | ***up*** |  | ***up*** | ***up*** |  | ***up*** |  |  |  |  |  | up |  |
| ***miR-155-5p*** |  |  |  | ***up*** | ***up*** |  |  | ***up*** |  | ***up*** |  |  |  |  | up |  |
| ***miR-224-5p*** |  |  |  | ***up*** | ***up*** |  | ***up*** |  |  |  |  |  |  | down | up |  |
| ***miR-34a-5p*** | ***up*** | ***up*** | ***up*** |  |  |  |  | ***up*** |  |  |  |  |  |  |  |  |
| ***miR-138-5p*** |  |  |  |  |  |  |  | ***down*** |  | ***down*** | ***down*** |  | up |  |  |  |
| ***miR-187-3p*** |  |  |  | ***up*** | ***up*** |  |  |  |  |  | ***up*** |  |  |  | up |  |
| ***miR-31-5p*** |  |  | ***up*** | ***up*** |  |  |  |  |  | ***up*** |  |  |  |  |  |  |
| ***miR-26a-5p*** |  |  | down |  |  |  |  | down | up |  |  |  | ***down*** | ***down*** |  | ***down*** |
| ***miR-125b-5p*** |  |  |  |  |  |  | up |  | up |  |  |  | ***down*** | ***down*** |  | ***down*** |
| miR-34b-5p | up | up |  |  |  |  |  |  |  | down |  |  |  |  |  |  |
| miR-21-5p |  |  | up |  |  |  |  |  | up |  |  |  | up |  |  | up |
| miR-29b-3p |  |  | up |  |  |  |  | up |  |  |  |  | down | down |  |  |
| miR-100-5p |  |  | down |  |  |  |  |  |  |  | down |  | down |  |  |  |
| miR-130a-3p |  |  | down |  |  |  |  |  |  |  | down |  | down |  |  |  |
| miR-15b-5p |  |  | down |  |  |  | down |  |  |  |  |  | down |  |  |  |
| miR-181a-5p |  |  |  |  |  |  | up | up |  |  |  |  | down |  |  |  |
| miR-199a-5p |  |  | down |  |  |  | down |  |  |  |  |  | down |  |  |  |
| miR-203a |  |  | up |  |  |  |  |  |  |  | up |  |  | down |  |  |
| miR-29c-3p | up |  |  |  |  |  |  | up |  |  |  |  | down |  |  |  |
| miR-7-5p |  | down | down |  |  |  |  |  |  |  |  |  | down |  |  |  |
| miR-144-5p |  | down |  |  |  |  |  |  |  |  | down |  |  |  |  |  |
| miR-15a-5p |  | up | up |  |  |  |  |  |  |  |  |  |  |  |  |  |
| miR-181c-5p |  |  |  |  |  |  | up | up |  |  |  |  |  |  |  |  |
| miR-183-5p | up |  |  |  |  |  |  |  |  |  | up |  |  |  |  |  |
| miR-213 |  |  |  |  |  |  | up | up |  |  |  |  |  |  |  |  |
| miR-21-3p |  |  |  |  |  |  |  | up |  |  | up |  |  |  |  |  |
| miR-220 |  |  |  |  |  |  | up | up |  |  |  |  |  |  |  |  |
| miR-221-5p |  | up |  |  |  |  |  |  |  |  | up |  |  |  |  |  |
| miR-222-5p |  |  |  |  |  |  |  | up |  |  | up |  |  |  |  |  |
| miR-345-5p |  |  | down |  |  |  |  | down |  |  |  |  |  |  |  |  |
| miR-34a-3p | up | up |  |  |  |  |  |  |  |  |  |  |  |  |  |  |
| miR-451a |  |  | down |  |  |  |  |  |  |  | down |  |  |  |  |  |
| miR-551b-3p |  | up |  |  |  |  |  |  |  |  | up |  |  |  |  |  |
| miR-7-2-3p |  | down |  |  |  |  |  |  |  |  | down |  |  |  |  |  |
| let-7d-5p |  |  |  |  |  |  |  |  |  |  | down |  | down |  |  |  |
| miR-130b-3p |  |  |  |  |  |  |  |  |  | down |  |  | up |  |  |  |
| miR-195-5p |  |  |  |  |  |  |  |  |  |  | down |  | down |  |  |  |
| miR-204-5p |  |  |  |  |  |  |  |  |  |  | down |  | down |  |  |  |
| miR-486-5p |  |  |  |  |  |  |  |  |  |  | down |  | down |  |  |  |
| miR-1 |  |  |  |  |  |  |  |  |  | down |  |  |  |  |  |  |
| miR-1179 |  |  |  |  |  |  |  |  |  |  | down |  |  |  |  |  |
| miR-1180 |  |  |  |  |  |  |  |  |  |  | down |  |  |  |  |  |
| miR-1249 |  |  |  |  |  |  |  |  |  |  | down |  |  |  |  |  |
| miR-1291 |  |  |  |  |  |  |  |  |  |  | down |  |  |  |  |  |
| miR-135b-5p |  |  |  |  |  |  |  |  |  |  | up |  |  |  |  |  |
| miR-138-1-3p |  |  |  |  |  |  |  |  |  |  | down |  |  |  |  |  |
| miR-146b-3p |  |  |  |  |  |  |  |  |  |  | up |  |  |  |  |  |
| miR-147b |  |  |  |  |  |  |  |  |  |  | up |  |  |  |  |  |
| miR-17-3p |  |  |  |  |  |  |  |  |  |  | up |  |  |  |  |  |
| miR-181b-3p |  |  |  |  |  |  |  |  |  |  | up |  |  |  |  |  |
| miR-182-5p |  |  |  |  |  |  |  |  |  |  | up |  |  |  |  |  |
| miR-197 |  |  |  |  | up |  |  |  |  |  |  |  |  |  |  |  |
| miR-20b-5p |  |  |  |  |  |  |  |  |  |  | down |  |  |  |  |  |
| miR-3200-3p |  |  |  |  |  |  |  |  |  |  | down |  |  |  |  |  |
| miR-33b-5p |  |  |  |  |  |  |  |  |  |  | up |  |  |  |  |  |
| miR-3913-5p |  |  |  |  |  |  |  |  |  |  | up |  |  |  |  |  |
| miR-455-3p |  |  |  |  |  |  |  |  |  |  | down |  |  |  |  |  |
| miR-486-3p |  |  |  |  |  |  |  |  |  |  | down |  |  |  |  |  |
| miR-532-5p |  |  |  |  |  |  |  |  |  |  | down |  |  |  |  |  |
| miR-574-3p |  |  |  |  |  |  |  |  |  |  | down |  |  |  |  |  |
| miR-585 |  |  |  |  |  |  |  |  |  |  | down |  |  |  |  |  |
| miR-589-5p |  |  |  |  |  |  |  |  |  |  | up |  |  |  |  |  |
| miR-652-3p |  |  |  |  |  |  |  |  |  |  | down |  |  |  |  |  |
| miR-744-3p |  |  |  |  |  |  |  |  |  |  | up |  |  |  |  |  |
| miR-873-5p |  |  |  |  |  |  |  |  |  |  | down |  |  |  |  |  |
| miR-891a |  |  |  |  |  |  |  |  |  |  | up |  |  |  |  |  |
