# Supplementary material for: Integrated Analysis of Thyroid Cancer Public Datasets Reveals Role of Post-Transcriptional Regulation on Tumor Progression by Targeting of Immune System Mediators
Source: PLoS One. 2015 Nov 4;10(11):e0141726. doi: 10.1371/journal.pone.0141726 (PMC4633176; doi:10.1371/journal.pone.0141726)
Supplement: S3 Table — (DOCX) [file pone.0141726.s004.docx]

**S3 Table.** Gene expression datasets selected for construction of regulatory network of post-transcriptional regulation.

| *Dataset* | *Tumor type* | *# Samples (Patients)* | *Platform* | *# unique genes* | *# Probes* | *GEO Series Number* | *Reference* |
| --- | --- | --- | --- | --- | --- | --- | --- |
| Tomas | PTC | 49 | GPL570 [HG- | 21,244 | 54,675 | GSE33630 | not available |
|  | ATC | 11 | U133_Plus_2] |  |  |  |  |
|  | Normal | 45 | Affymetrix |  |  |  |  |
| He | PTC and matched normal tissue | 9 | GPL570 [HG-U133_Plus_2] Affymetrix | 21,244 | 54,675 | GSE3467 | He H et al, 2005 |
| Salvatore | PTC | 10 | GPL5917 Human | 9,387 | 12,483 | GSE9115 | Salvatore G et al*,* 2007 |
|  | ATC | 5 | 12K cDNA clones |  |  |  |  |
|  | Normal | 4 |  |  |  |  |  |
| Giordano | PTC | 26 | GPL96 [HG- | 13,208 | 22,284 | GSE27155 | Giordano TJ et al, 2005 |
|  | ATC | 4 | U133A] Affymetrix | |  |  |  |
|  | FTC | 13 |  |  |  |  |  |
|  | Normal | 4 |  |  |  |  |  |
| Reyes | PTC and matched normal tissue | 7 | GPL570 [HG-U133_Plus_2] Affymetrix | 21,244 | 54,675 | GSE3678 | not available |
