# Supplementary material for: Integrated Analysis of Thyroid Cancer Public Datasets Reveals Role of Post-Transcriptional Regulation on Tumor Progression by Targeting of Immune System Mediators
Source: PLoS One. 2015 Nov 4;10(11):e0141726. doi: 10.1371/journal.pone.0141726 (PMC4633176; doi:10.1371/journal.pone.0141726)
Supplement: S4 Table — (DOCX) [file pone.0141726.s005.docx]

**S4 Table.** Gene Set Enrichment Analysis of thyroid cancer transcriptome.

|  |  | | | |
| --- | --- | --- | --- | --- |
| Enriched pathways | Gene count | % | fold | p-value |
| PTC | 2094 |  |  |  |
| *ECM-receptor interaction* | 21 | 1.06 | 1.93 | *0.004155* |
| *p53 signaling pathway* | 18 | 0.90 | 2.05 | *0.004698* |
| *Focal adhesion* | 37 | 1.86 | 1.42 | *0.023393* |
| *Regulation of actin cytoskeleton* | 38 | 1.91 | 1.37 | *0.037932* |
| *MAPK signaling pathway* | 45 | 2.26 | 1.30 | *0.048028* |
| TGF-beta signaling pathway | 18 | 0.90 | 1.60 | 0.050051 |
| ATC | 2749 |  |  |  |
| *ECM-receptor interaction* | 29 | 1.10 | 1.85 | *0.000871* |
| *Focal adhesion* | 52 | 1.98 | 1.39 | *0.009773* |
| *p53 signaling pathway* | 29 | 1.10 | 2.29 | *1.21E-05* |
| *Adherens junction* | 22 | 0.84 | 1.53 | *0.039474* |
| *Cell cycle* | 52 | 1.98 | 2.23 | *4.77E-09* |
| *Gap junction* | 27 | 1.03 | 1.63 | *0.009712* |
|  |  |  |  |  |
